# Synchrotron X-ray induced acoustic imaging

**DOI:** 10.1038/s41598-021-83604-3

**Published:** 2021-02-18

**Authors:** Seongwook Choi, Eun-Yeong Park, Sinyoung Park, Jong Hyun Kim, Chulhong Kim

**Affiliations:** 1 Department of Electrical Engineering and Creative IT Engineering, Medical Device Innovation Center, Pohang University of Science and Technology, Pohang, Republic of Korea; 2 Department of Mechanical Engineering, Pohang University of Science and Technology, Pohang, Republic of Korea; 3 Pohang Accelerator Laboratory, Pohang, Republic of Korea

**Keywords:** Photoacoustics, Biomedical engineering, Imaging and sensing

## Abstract

X-ray induced acoustic imaging (XAI) is an emerging biomedical imaging technique that can visualize X-ray absorption contrast at ultrasound resolution with less ionizing radiation exposure than conventional X-ray computed tomography. So far, medical linear accelerators or industrial portable X-ray tubes have been explored as X-ray excitation sources for XAI. Here, we demonstrate the first feasible synchrotron XAI (sXAI). The synchrotron generates X-rays, with a dominant energy of 4 to 30 keV, a pulse-width of 30 ps, a pulse-repetition period of 2 ns, and a bunch-repetition period of 940 ns. The X-ray induced acoustic (XA) signals are processed in the Fourier domain by matching the signal frequency with the bunch-repetition frequency. We successfully obtained two-dimensional XA images of various lead targets. This novel sXAI tool could complement conventional synchrotron applications.

## Introduction

X-ray induced acoustic imaging (XAI) is an emerging imaging technique that takes advantage of high ultrasound (US) resolution and sensitive X-ray absorption contrast^1–7^. XAI has shown great potential for low-dose 3D-imaging and non-invasive and real-time dosimetry^8–14^. In XAI, X-ray induced acoustic (XA) waves are generated through rapid thermoelastic expansion when X-ray absorbing materials receive pulsed X-ray irradiation. The pressure of the XA waves can be formulated as follows^15–21^:

1 $$\left( {\nabla^{2} - \frac{1}{{v_{s}^{2} }}\frac{\partial }{{\partial t^{2} }}} \right)p\left( {\overrightarrow {{r_{0} }} ,t} \right) = - \frac{\beta }{{C_{p} }}\frac{{\partial H\left( {\overrightarrow {{r_{0} }} ,t} \right)}}{\partial t},$$

where ∇ represents the gradient, $p\left( {\overrightarrow {{r_{0} }} ,t} \right)$ is the initial pressure rise of the XA wave at location $\overrightarrow {{r_{0} }}$ and time $t$, $v_{s}$ is the speed of sound, $\beta$ is the thermal coefficient of volume expansion, $C_{p}$ is the heat capacity, and $H\left( {\overrightarrow {{r_{0} }} ,t} \right)$ is the heating function. When the delta function excites the absorbing material, the initial pressure $p_{0}$, rises:

2 $$p_{0} = {\Gamma } \times \eta_{th} \times \mu \times F,$$

where ${\Gamma } = \beta v_{s}^{2} /C_{p}$ is the Gruneisen parameter, $\eta_{th}$ is the proportion of incident energy that is absorbed, $\mu$ is the X-ray absorption coefficient, and $F$ is the X-ray fluence. The amplitudes of the XA waves are linearly proportional to the X-ray absorption coefficients, and thus XAI can be used for real-time dosimetry. The XA waves propagate omnidirectionally and are detected by conventional US transducers. If arrays of US transducers are used, 2D or 3D tomographic images can be obtained with single X-ray irradiation. Consequently, XAI requires much less ionizing radiation exposure than conventional imaging methods, such as X-ray computed tomography (CT). Until recently, XAI has been actively conducted. Elijah et al. demonstrated the feasibility of bone imaging using XAI, and Yang et al. presented the simulation study of 3D bone imaging with a spherical probe^4,22^. Donghyun et al. performed 3D imaging with a lead target and Wei et al. developed dual-modality XA and ultrasound imaging for real-time monitoring of radiotherapy^8,9^.

Thus far, medical linear accelerators (LINACs) or industrial portable X-ray tubes have been adapted as X-ray excitation sources for XAI^23,24^. A medical LINAC typically has a pulse width of several micro seconds, which is too long to generate MHz acoustic signals. Furthermore, it is difficult to control the parameters of a LINAC, such as the beam size, pulse width, and pulse repetition rate. More importantly, the energy band of therapeutic medical LINACs is not suitable for imaging purposes because tissue X-ray absorption is relatively low in this energy band. As an alternative to a LINAC, industrial portable X-ray tubes, widely used for non-destructive testing, have been tested for XAI. These produce energy in a range similar to that of diagnostic X-ray imaging, with a pulse width of tens of nanoseconds, suitable for generating MHz US signals. However, these portable X-ray tubes suffer from short lifespans and are unstable. In addition, their X-ray excitation intensity is relatively low resulting in the low signal-to-noise ratios (SNR).

In this letter, we demonstrate the feasibility of a novel XAI system adapted to use a synchrotron, which can overcome the limitations of conventional sources. We acquired XA signals from lead samples by analyzing the raw data in the frequency domain, and then we performed 2D XA imaging of various lead targets.

## Results

### Synchrotron X-ray induced acoustic imaging system

**Figure 1:**
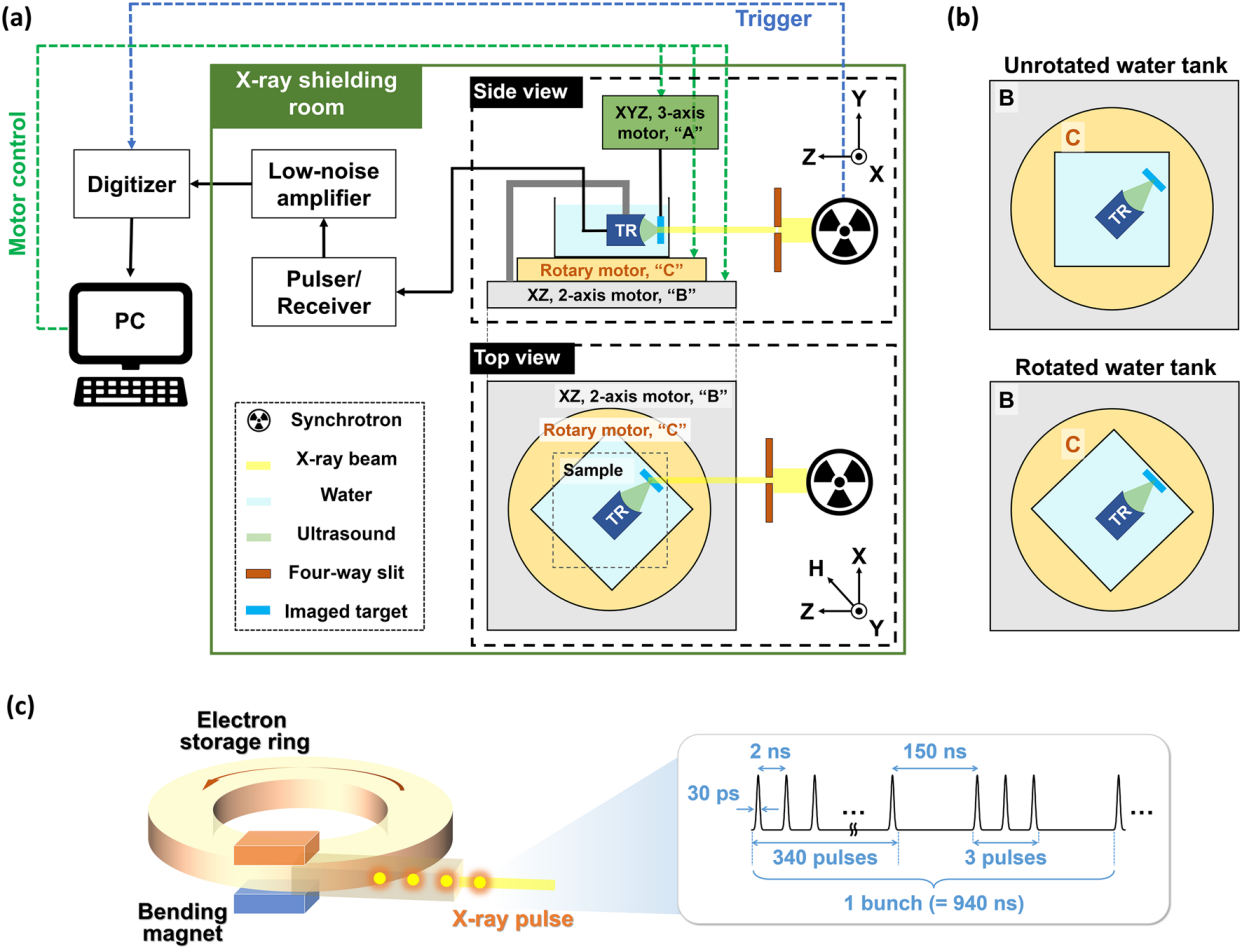
(**a**) Schematic of a synchrotron X-ray induced acoustic imaging system. (**b**) Alignment of an imaging target, ultrasound transducer, and water tank. (**c**) Schematic of the X-ray synchrotron and the X-ray pulse sequence; TR, transducer.

Figure 1a is a schematic of the synchrotron X-ray induced acoustic imaging system (sXAI). The X-ray beam is extracted from the 9D white-beam beamline of the Pohang Light Source (PLS)-II, Republic of Korea. First, electrons are injected from a linear accelerator into a storage ring. Then a bending magnet based X-ray beam is directed into the chamber^25^. The X-ray source from the 9D beamline has a dominant energy of 4 to 30 keV, a pulse repetition period of 2 ns, and a bunch-repetition period of 940 ns (Fig. 1c). Each bunch consists of 343 pulses, with a single pulse width of 30 ps. The cross-sectional size of the pulsed X-ray is adjusted by a four-way slit aperture. The pulsed X-ray is delivered to an imaging target to generate XA signals. To detect the XA signals, we used a single-element focused US transducer (KPS100-1-P38, The Ultran Group, USA) with a center frequency of 1 MHz, an aperture size of 25 mm, and a focal length of 38 mm. The US transducer and X-ray absorbing target are immersed in a polypropylene water tank for acoustic impedance matching. The pulser/receiver (5073PR, Olympus NDT Inc., USA) and low-noise amplifier (SR560, Stanford Research Systems, USA) improve the signal amplitudes. A high-speed digitizer (ATS9350, Alazar Technologies Inc., Canada) is used for data acquisition. Three motorized stages in the 9D beamline are used to align the system and scan a target (Fig. 1a). The first stage, “A”, is an XYZ-axis motorized stage, and an imaging target is fixed to this stage to be moved for raster scanning. The second stage, “B”, is an XZ-axis motorized stage, which is used to align the imaging target and US transducer with the excitation X-ray beam. Finally, “C” is a rotary stage that rotates the water tank to control the length of the water path from the water tank wall to the imaged target (Fig. 1b).

**Figure 2:**
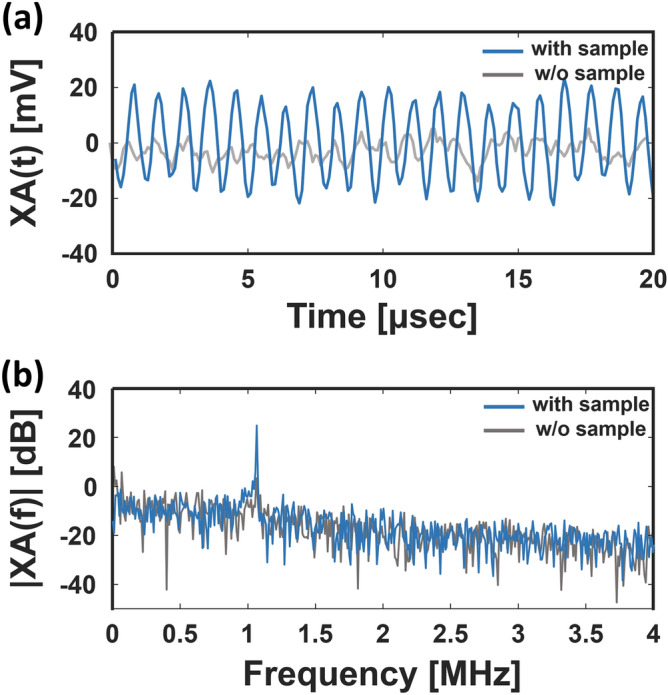
(**a**) X-ray induced acoustic (XA) signal in the time domain with and without the target. (**b**) XA signal in the frequency domain.

Although XA signals are generated upon X-ray irradiation, it is difficult to separate successive XA signals generated by single pulses because the pulse-repetition period of the synchrotron is so much shorter (e.g., 2 ns) than the propagation time of acoustic waves (e.g., 1 μs for a propagation distance of 1.5 mm in water). Therefore, the XA effect in our system is induced by using a bunch of X-ray pulses rather than a single pulse. As shown in Fig. 2a, the XA signals are not distinguishable in the time domain. After fast Fourier transform (FFT), the XA signals are dominant at a frequency of 1.064 MHz, the matching frequency of one X-ray beam, in the frequency domain (Fig. 2b).

### Comparison of X-ray induced acoustic signals

**Figure 3:**
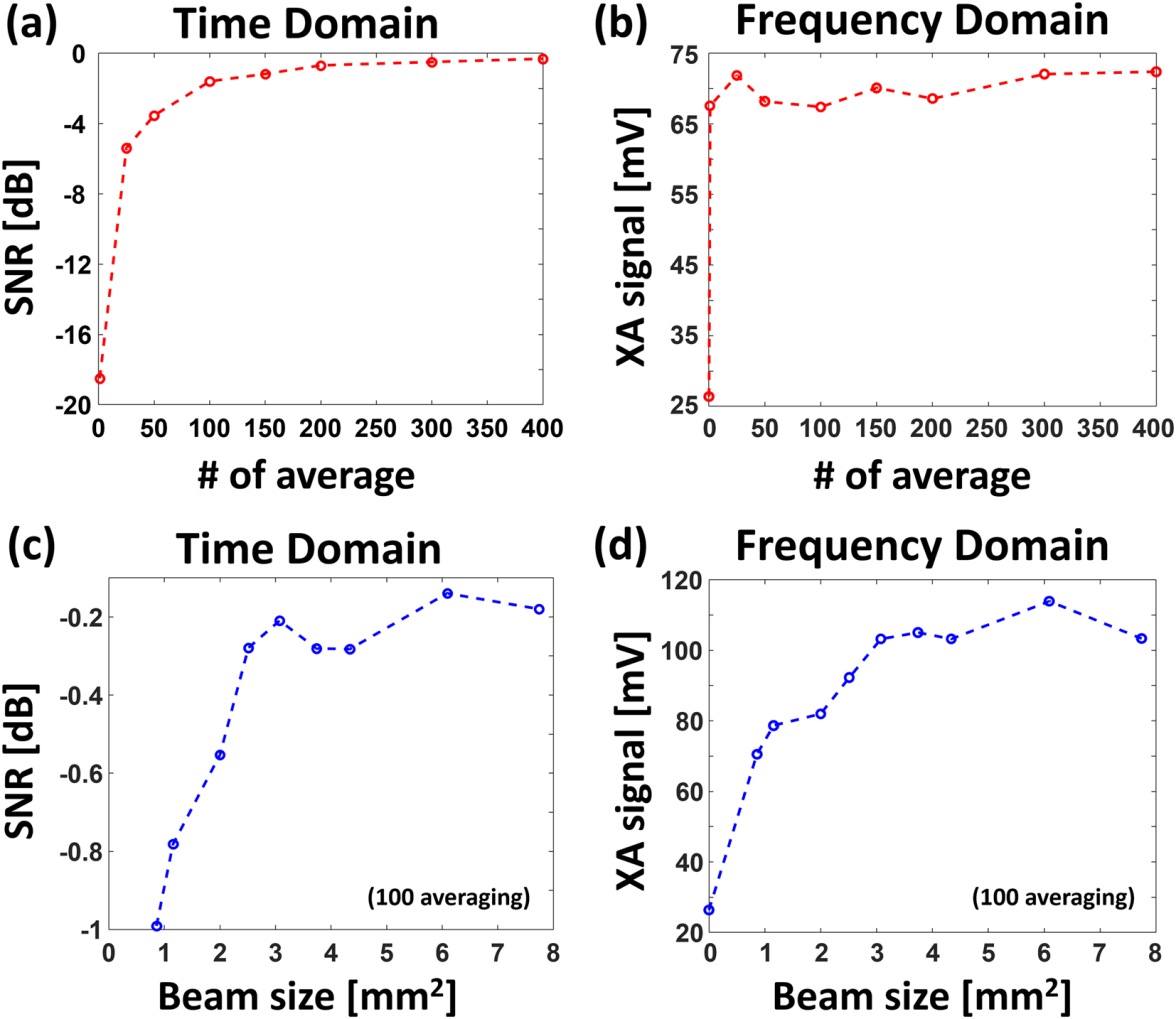
(**a**) Signal-to-noise ratios (SNRs) vs. the number of averages in the time domain. (**b**) XA signal amplitudes vs. the number of averages in the frequency domain. (**c**) SNRs vs. X-ray beam sizes in the time domain. (**d**) XA signal amplitudes vs. X-ray beam sizes in the frequency domain.

As an initial experiment, we compared the averaged signal of the XA signals in both the time and frequency domains. As seen in Fig. 3a, the value of SNR_time_ initially increases but begins to saturate after the average count of 100. It is difficult to identify XA signals with fewer than 50 averages in the time domain. On the other hand, the XA signal amplitudes can be distinguished regardless of the average count in the frequency domain, because the XA signals are periodic but the noises are not (Fig. 3b). This result implies that sXAI can be performed without averaging in the frequency domain, although the time-domain signals suffer from a low SNR, a fundamental limitation of conventional X-ray acoustic imaging systems.

Next, we compared the XA signals in the both time and frequency domains with respect to the X-ray beam size. We set the average count of XA signals to be 100, and set the other parameters (e.g., the sampling frequency, record length, and gain) to be the same as in the above setup. The SNRs in the time domain and XA signal amplitudes in the frequency domain initially increase, but begin to saturate beyond an X-ray beam size of 3.1 mm^2^ (Fig. 3c,d). The main reason is that this area is approximately the maximum overlap region between the X-ray beam and US focal spot size (e.g., 3.3 mm^2^).

### Measurement of spatial resolutions

**Figure 4:**
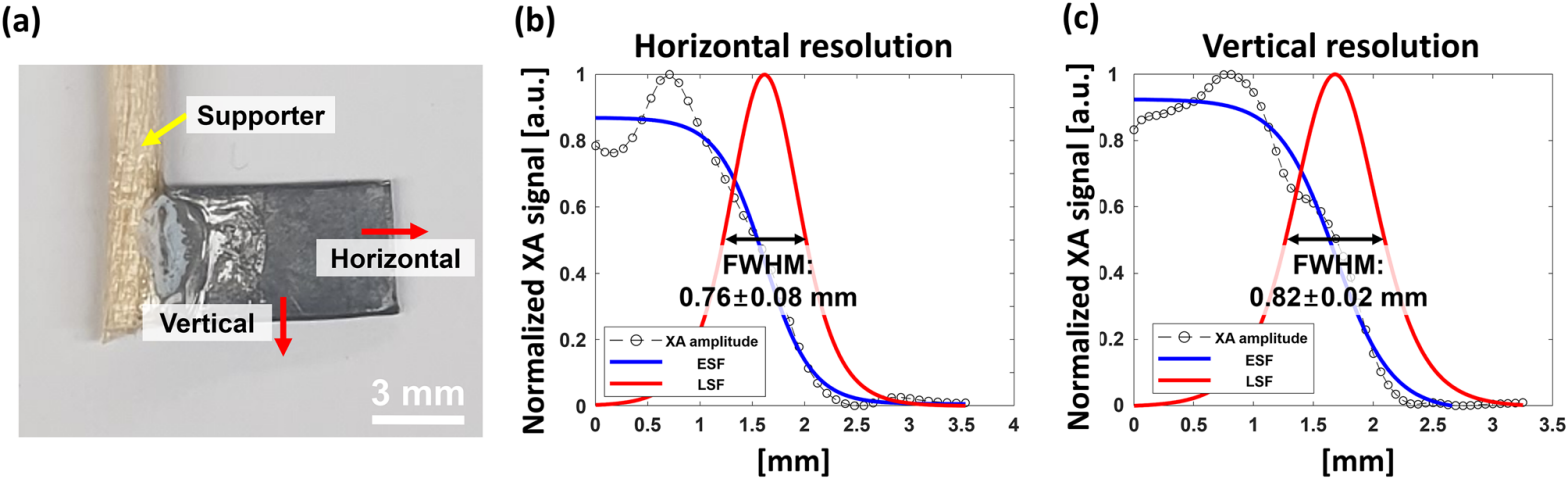
(**a**) Photograph of a rectangular lead target. ESF and LSF fittings from experimental XA data across (**b**) the horizontal and (**c**) the vertical directions; XA, X-ray induced acoustic; FWHM, full width at half maximum; ESF, edge spread function; and LSF, line spread function.

To measure the lateral resolution of the sXAI system, we imaged a rectangular lead target with a thickness of 1 mm (Fig. 4a). The step sizes along the H axis and Y axis were set to be 0.35 mm and 0.25 mm, respectively. Moreover, the X-ray beam size was 2.5 mm^2^. Initially, we obtained the edge spread functions (ESFs) of the XA image in horizontal (H axis) and vertical (Y axis) directions, and plotted the associated line spread functions (LSFs). Then, the full widths at half maxima (FWHMs) of the LSFs were defined as the lateral resolutions. Note that the XA signals within the lead target fluctuate considerably due to the uneven surface of the lead sample. The estimated horizontal and vertical resolutions are 0.76 ± 0.08 and 0.82 ± 0.02 mm, respectively (Fig. 4b,c).

### X-ray induced acoustic 2D imaging

**Figure 5:**
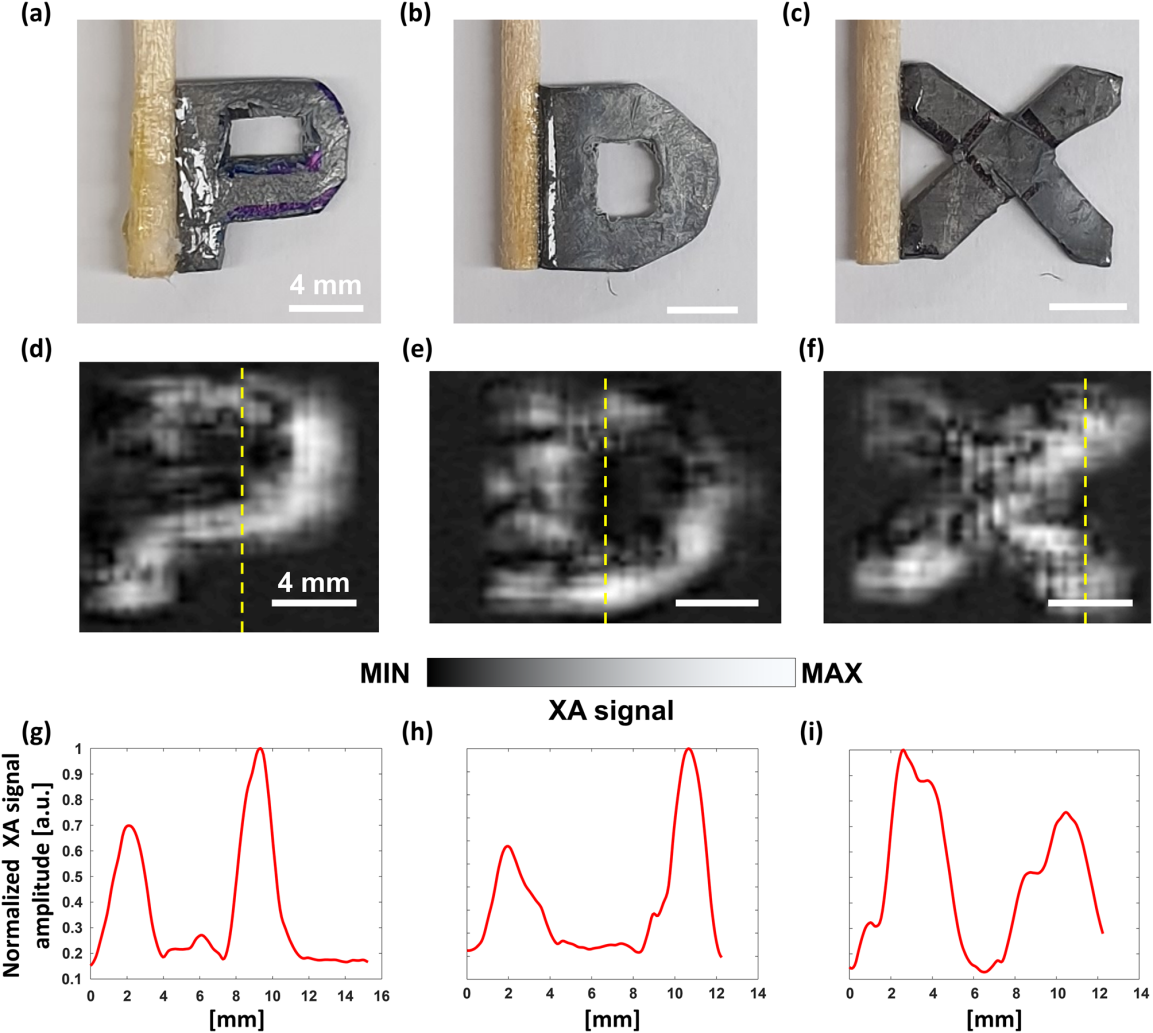
(**a**–**c**) Photographs of the three lead letter targets, “P”, “D”, and “X”, and (**d**–**f**) the associated X-ray induced acoustic images, respectively. (**g**–**i**) Line profiles acquired along the yellow dotted lines in (**d**–**f**), respectively.

We performed 2D imaging of three lead targets in the shapes of the letters “P”, “D”, and “X” (Fig. 5a-c). The step sizes along the H and Y axes were the same as in the above experiments. For imaging the letter “P”, there were 40 steps in the horizontal direction (H axis) and 61 steps in the vertical direction (Y axis). For imaging the letters “D” and “X”, the vertical (Y axis) step counts were both 49, but the horizontal (H axis) step counts were 48 and 44, respectively. For post image processing, cubic interpolation was employed, so the image pixels were quadrupled in both the horizontal and vertical directions. We successfully acquired the XA images of the three letters as shown in Figs. 5d-f, and the XA images match well with the photographs (Figs. 5a-c). However, the lead material has low stiffness then it could not be completely flat. Therefore, the surface roughness of the lead samples makes the distribution of XA signals uneven in the XA images. The line profiles obtained along the dotted yellow lines in Figs. 5d-f are shown in Figs. 5g-i, respectively.

## Discussion

In summary, we demonstrated the feasibility of a novel XAI system adapted to use a synchrotron, called sXAI. We successfully obtained 2D XA images of three letter-shaped lead targets. Conventional XAI systems use medical LINACs or portable industrial X-ray tubes, which have limited energy ranges, pulse widths, and lifespans. A synchrotron can potentially overcome these limitations, but our current system poses several challenges. First, because we cannot control the X-ray pulse repetition rate, we must analyze the XA signals in the frequency domain, using the bunch-repetition frequency. Consequently, we obtain the XA signals without averaging and process the signals in the frequency domain, unlike conventional XAI systems, which require a number of averages in the time domain due to low SNR. In this case, however, it is difficult to acquire the temporal resolution along the depth direction. PAL can be temporarily tested using the machine study time to operate one electron pulse rather than 343 electron pulses, then we can perform 3D synchrotron XAI. In this case, the storage ring's RF timing system allows only one electron pulse to run on the storage ring by synchronizing the electron bundle with the RF bucket. Second, spatial resolution is currently determined by the overlapped area between the X-ray and US beams. Hence, we need to reduce the US beam size or the X-ray beam size to increase the spatial resolution. To decrease the X-ray beam size, we can utilize X-ray focusing technologies, such as a capillary lens or Kirkpatrick-Baez mirror^26,27^. Then, the spatial resolution can potentially be significantly improved. Finally, the novel sXAI technique could complement conventional synchrotron application. The synchrotron, including PAL, is also utilized for imaging and analyzing nano-structured polymer, macromolecule, nano compound, amorphous substance, etc. in the range of 4 to 30 keV. It also performs biomedical imaging with small animals about muscles, bones, and brains in this energy range. In the future, the sXAI can be equipped as dual modality with conventional synchrotron application.

## Methods

### Experiment procedures

For all experiments, a 1 mm thick lead target was used as an X-ray absorbing material due to its high X-ray absorption coefficient. Positioning the target and aligning the system are detailed as follows: First, the target is placed at the focal point of the US transducer by checking the US echo intensity. To protect the US transducer from radiation damage, it should not be placed normal to the X-ray beam path, so the transducer and the target are placed at an angle to the beam path. Then, we attach a burn paper (Type 2167 PX-2870, Kodak, USA) to the front wall of the water tank, where the X-ray is aimed, to verify the irradiated location and beam size. When the burn paper turns black, it confirms the X-ray irradiation. We use the XYZ-axis stage “A” and the XZ-axis stage “C” to align the target with the X-ray beam. Additionally, we rotate the water tank 45° relative to the X-ray beam path, positioning the target parallel to the water tank wall (Fig. 1b). In this geometry, the interaction path length between water and X-ray beam along the target surface is constant to achieve constant water attenuation. This ensures that all pixels of the target have a constant signal-to-noise ratio in 2D scanning. We define an axis rotated 45° from the X axis to the Z axis as the “H” axis, meaning that the imaging target is placed on the HY plane (Fig. 1a). For 2D imaging, the imaging target moved for scanning. In horizontal scanning along the H axis, one step in the horizontal direction is achieved by moving one step along the X-axis and one step along the Z-axis. For vertical scanning, one step in the vertical direction is simply one step along the Y-axis. Thus, the 2D scanning workflow is to move up a specified number of steps vertically, move one step in the horizontal direction, move down stepwise in the vertical direction, then move one step in the horizontal direction, and repeat the whole procedure.

### Comparison of X-ray induced acoustic signals

We set the beam size as 2.6 mm^2^ (2.0 mm × 1.3 mm along H axis × Y axis, respectively), the sampling frequency as 5 MS/s, the record length as 4096 samples, the gain of pulser/receiver as 44 dB, and the gain of the low-noise amplifier as 500. To compare the XA signals in the time domain, we defined SNR_time_ as

3 $${\text{SNR}}_{{{\text{time}}}} { }\left[ {{\text{dB}}} \right] = 20log\left( {\frac{A\left( i \right)}{{rms\left( {s\left( i \right)} \right)}}} \right),$$

where *s*(*i*) is the averaged XA-signal dataset of the *i*th experiment, *rms*(*x*) is the root mean square of dataset *x*, and *A*(*i*) is the XA signal amplitude of *s*(*i*).
